# The effect of elevated CO_2_ on hyperspectral leaf reflectance in mature trees

**DOI:** 10.1007/s00468-025-02650-w

**Published:** 2025-07-10

**Authors:** Anna Lee Jones, Anna Gardner, Felicity Hayes, Christian Pfrang, Elizabeth S. Jeffers

**Affiliations:** 1 Department of Biology, University of Oxford, South Parks Road, Oxford, OX1 3SZ UK; 2 School of Biological Sciences, University of Birmingham, Birmingham, B15 2TT UK; 3 Birmingham Institute of Forest Research (BIFoR), University of Birmingham, Birmingham, B15 2TT UK; 4 UK Centre for Ecology and Hydrology, Environment Centre Wales, Deiniol Road, Bangor, LL57 2UW UK; 5 School of Geography, Earth and Environmental Sciences, University of Birmingham, Birmingham, B15 2TT UK; 6 Department of Plant Sciences, University of Cambridge, Cambridge, UK

**Keywords:** Climate change, Forests, Leaf reflectance, Spectroscopy, Vegetation indices, Forest health

## Abstract

Experimentally elevated CO_2_ does not significantly alter the overall leaf reflectance of mature *Quercus robur L.*, but increases Plant Senescence Reflectance Index (PSRI) suggesting a change in the ratio of chlorophyll to carotene content.

Rising atmospheric CO_2_ concentrations, driven by anthropogenic emissions, are projected to reach 550 ppm by 2050. Elevated CO_2_ (eCO_2_) is expected to have a fertilisation effect on forests, influencing productivity, water relations, and phenology. However, the impact of eCO_2_ on leaf reflectance in mature forests remains poorly understood, despite its critical role in radiative transfer processes and remote sensing of forest health. Utilising the Birmingham Institute of Forest Research (BIFoR) Free-Air CO_2_ Enrichment (FACE) experiment, we investigated the hyperspectral leaf reflectance of 180-year-old *Quercus robur* L. trees exposed to eCO_2_ for 7 years. Our results demonstrate that overall leaf reflectance under eCO_2_ is similar to that of leaves exposed to ambient CO_2_, but the Plant Senescence Reflectance Index (PSRI) is significantly higher under eCO_2_. This index relates to the ratio of foliar chlorophyll and carotene pigments. These findings suggest that *Q. robur* reflectance will not significantly shift under future CO_2_ conditions, but the relative content of pigments will change, altering the reflectance of specific wavelengths and providing insights into the leaf level physiological and phenological responses of mature trees to eCO_2_.

## Introduction

Since 1850, net anthropogenic CO_2_ emissions have reached 2400 (± 240) Gt, with concentrations in 2019 surpassing levels observed over the past two million years (Calvin et al. 2023). Under the ‘business as usual’ RCP 8.5 scenario, atmospheric CO_2_ concentrations are predicted to reach 550 ppm by 2050 (IPCC 2023). Forests are a major component of the terrestrial carbon sink, absorbing 2.4 (± 0.4) petagrams of carbon per year (Pg C year^−1^) globally (Pan et al. 2024). CO_2_ is the carbon input for photosynthesis in plants and elevated CO_2_ (eCO_2_) has a stimulating effect on photosynthesis, often referred to as the ‘CO_2_ fertilisation’ effect (Ciais et al. 2013). CO_2_ fertilisation of forests, leading to enhanced productivity, has been observed in recent decades (Zhu et al. 2016; Ruehr et al. 2023). However, the long-term effect of eCO_2_ on mature forests and their role as a carbon sink remains uncertain.

The fertilisation response of tree seedlings and saplings to eCO_2_ has been extensively experimentally studied using Free-Air CO_2_ Enrichment (FACE) experiments (as reviewed by Ellsworth et al. 2004; Ainsworth and Long 2005; Maschler et al. 2022). The first generation of FACE experiments provided valuable long-term observations of the effect of eCO_2_ on young trees, such as sustained elevated photosynthetic rate (Ainsworth and Long 2005) and increased fine-root production and throughput of root biomass into the soil (Norby and Zak 2011). However, these experiments were conducted in young tree plantations, limiting their application to understanding how mature forests will respond to eCO_2_ (Norby et al. 2005; U.S. DOE 2020). Notably, results from the Aspen FACE project revealed that responses observed in seedlings and young trees were not always sustained as the stand matured, emphasising the effect of tree age on eCO2 responses (Burton et al. 2014). Wood analysis of *Quercus ilex* exposed to a lifetime of eCO_2_ (at a natural CO_2_ spring) revealed that most growth enhancement occurs when the tree is young (Hättenschwiler et al. 1997), leading to ongoing questions about the growth responses of mature forests to eCO_2_.

The second generation of FACE experiments seeks to explore the capacity of mature trees to response to eCO_2_ across different biomes (Norby et al. 2016). This study is based at BIFoR FACE, situated in a 180-year-old *Quercus robur* (*Q. robur*) dominated woodland in central England, which has shown increased photosynthetic activity and woody biomass production in the first 7 years of eCO_2_ exposure (Gardner et al. 2022a; Norby et al. 2024). These results contrast with the Eucalyptus FACE experiment (EucFACE) based in Australia, where increased carbon uptake did not translate into greater carbon sequestration, likely due to phosphorous limitation (Jiang et al. 2020). As well as the eCO_2_ crane experiment in Basel, where after 8 years of eCO_2_ treatment, radial stem diameter was not affected in *Quercus petraea* or the other deciduous hardwoods tested (Bader et al. 2013).

In addition to changes in photosynthetic rate and tree growth, eCO_2_ has also been shown to affect water relations in trees. In a synthesis of 13 long-term experiments (> 1 year), Medlyn et al. found European forest trees exposed eCO_2_ had 21% lower stomatal conductance (Medlyn et al. 2001), although a recent meta-analysis has shown that the eCO_2_ induced increase in water use efficiency (WUE) was primarily due to increased photosynthesis rather than decreased stomatal conductance (Gardner et al. 2023). eCO_2_ has been shown to increase WUE in mature deciduous forests (Leuzinger and Körner 2007) and *Quercus petraea* saplings (Ofori-Amanfo et al. 2020). After 8 years of eCO_2_ treatment, water use was significantly reduced in the Basel deciduous forest experiment (Bader et al. 2013), but at BIFoR FACE although tree’s water use was lower on average under eCO2 this change was not significant (Quick et al. 2025).

At the leaf level of trees, eCO_2_ has been shown to decrease chlorophyll content. Onisch et al. reported decreased chlorophyll in young *Picea* and *Fagus* saplings exposed to eCO_2_ (Onisch et al. 2016); and Tausz et al. found the needles of young *Picea abies* trees had lower chlorophyll when exposed to eCO_2_ (Tausz et al. 1996). In mature, forest-grown trees at the EucFACE experiment mature *Eucalyptus tereticornis* leaves had reduced pigment concentrations (chlorophyll and carotenes) per area (Wujeska-Klause et al. 2019). In mature *Liquidambar styraciflua* (sweetgum) leaves from the Oak Ridge FACE experiment, after 12 years of eCO_2_ exposure leaves had lower chlorophyll (Warren et al. 2015). In the BIFoR FACE experiment, eCO_2_ did not affect mass-based chlorophyll concentrations in mature *Q. robur* leaves, but area-based chlorophyll content significantly increased, which was attributed to increased leaf mass per unit area (Gardner et al. 2022b). Similarly, mature *Quercus ilex* and *Quercus pubescens* trees exposed to eCO_2_ by a natural spring did not show a significant change in the chlorophyll content of leaves (Schwanz and Polle 1998a).

ECO_2_ has been reported to also decrease the nitrogen content of leaves per unit area (Ellsworth et al. 2004; Ainsworth and Long 2005). In mature *Liquidambar styraciflua* trees, foliar nitrogen decreased 17% under long-term eCO_2_ (Warren et al. 2015). Reduced foliar nitrogen was also found in *Populus tremuloides*, *Betula papyrifera*, and *Acer saccharum* saplings (Agrell et al. 2000). It has been indicated that reduced foliar allocation of nitrogen to chlorophyll under eCO_2_ follows total nitrogen content (Warren et al. 2015; Wujeska-Klause et al. 2019). ECO_2_ has resulted in reduced foliar phenol content in *Quercus* species (Tognetti and Johnson 1999; Watanabe et al. 2021), although some studies have found this effect is only temporary (Dury et al. 1998). In the BIFoR FACE experiment, no significant effect of eCO_2_ on the foliar nitrogen content of *Q. robur* foliar has been found (Gardner et al. 2022b).

Studies have also examined the effect of eCO_2_ on leaf nutrient content, from the perspective of food sources for insects. In a range of deciduous species, saplings grown under eCO_2_ have reduced foliar nutritional quality (Dury et al. 1998). Carbohydrate concentrations, particularly starch, increase in young trees exposed to eCO_2_ (Kinney et al. 1997; Agrell et al. 2000; Coley et al. 2002). Combined with reduced nitrogen content, this results in increased foliar carbon to nitrogen (C:N) ratios. For example, at the BIFoR FACE experiment, the foliar carbon content of fresh leaves was found to be significantly higher under eCO_2_ (Roberts et al. 2022).

ECO_2_ also decreases the antioxidant content of tree leaves. For instance, in mature Mediterranean *Quercus* trees, eCO_2_ reduced antioxidant enzyme activity and increased the ascorbate pool (Schwanz and Polle 1998b; Marabottini et al. 2001). In *Q. robur* seedlings, superoxide dismutase content decreased under eCO_2_ (Schwanz et al. 1996) but this has not been investigated in mature *Q. robur* trees. In summary, eCO_2_ has been shown to lead to a variety of effects on the phytochemistry of deciduous trees, and on oak trees in particular although there is more limited evidence for the effects on mature trees in forest settings.

Remote sensing of forest health, productivity, and biodiversity relies on the reflectance spectra of leaves (Lausch et al. 2016; Kacic and Kuenzer 2022). Across visible to infrared wavelengths, leaf reflectance is influenced by factors such as pigments, leaf structural composition, and water content (Grant 1987; Baldini et al. 1997; Sims and Gamon 2002). Despite its importance, few studies have examined the effect of eCO_2_ on the leaf reflectance of mature trees, which could provide important insight into leaf-level changes underpinning forest fertilisation or acclimation. Hyperspectral reflectance data from forests are widely used to remotely sense leaf traits (Chen et al. 2022), classify species (Puttonen et al. 2010), and monitor forest health (Sonobe and Wang 2017; Torres et al. 2021) under natural conditions. Understanding how leaf reflectance will change under future CO_2_ concentrations is, therefore, crucial for accurately monitoring forest health and productivity in a changing climate.

Leaf-level hyperspectral reflectance studies on trees exposed to eCO_2_ are limited and have found variable results. Thomas (2005) found an increase in the reflectance of photosynthetically active radiation (PAR; 400–700 nm) and a decrease in chlorophyll in a glasshouse eCO_2_ study on tropical *Leguminosae* saplings (Thomas 2005). In contrast, Carter et al. (2000) found no significant effect of eCO_2_ on the reflectance of young *Acer saccharum* within a 400–850 nm range in open-trop chambers (Carter et al. 2000). In mature *Quercus pubescens* trees exposed to lifetime eCO_2_ via natural springs, normalised difference vegetation index (NDVI) was not affected and nor was Photochemical Reflectance Index (PRI) despite photosynthetic enhancement (Stylinski et al. 2000). Similarly, the Oak Ridge FACE experiment reported no significant changes in reflectance at the leaf or canopy level for a *Liquidambar styraciflua* monoculture under eCO2 (Wicklein et al. 2012). In the EucFACE experiment, harvested leaf reflectance in the PAR spectrum (400–700 nm) was not significantly affected by eCO_2_ treatment (Wujeska-Klause et al. 2019). However, Pintó-Marijuan et al. successfully used near-infrared (NIR; 1100–2500 nm) reflectance spectroscopy to examine pigment and antioxidant responses of *Quercus ilex* resprouts under eCO_2_ (Pintó-Marijuan et al. 2013). Our study aims to expand on these findings by examining a broad continuous range of hyperspectral reflectance (350–2500 nm) of leaves measured in situ in a mature *Q. robur* forest under eCO_2_.

The aim of our study is to investigate whether long-term eCO_2_ changes the leaf reflectance of mature *Q. robur* trees in terms of total reflectance, spectral profile, and vegetation indices. The majority of the relevant literature indicates eCO_2_ does not significantly affect the leaf reflectance of trees exposed over long time periods, particularly in mature trees. We, therefore, hypothesise that eCO_2_ will not significantly affect the leaf reflectance profile or total reflectance but may affect specific regions linked to physiological or structural changes. By examining the entire wavelength range reflectance profile as well as traditional vegetation indices, we expect to find more nuanced changes in leaf reflectance.

## Methods

### Study site

The Birmingham Institute of Forest Research (BIFoR) has operated a Free-Air Carbon Enrichment (FACE) experiment since 2015 at Mill Haft, Staffordshire, UK (52°48′3.6″ N, 2°18′0″ W). Mill Haft is 19.1 ha of deciduous woodland dominated by *Q. robur* (pedunculate oak) in the canopy and *Corylus avellana* (common hazel) in the understory, situated in a temperate maritime climate (Hart et al. 2020; MacKenzie et al. 2021). The underlying geology is Helsby Sandstone, and the dominant soil type is Dystric Cambisol, with a sandy clay texture (Norby et al. 2024). The long-term average annual temperature of the site is 9 °C, and the average annual rainfall is 690 mm (Norby et al. 2016). Since the CO_2_ fertilisation experiment began in 2017, mean hourly rainfall has been recorded by four rain gauges on the BIFoR FACE meteorological towers (TR-525M, Texas Electronics, Dallas, Texas), alongside daily mean air temperature (HMP155RH, Vaisala, Helsinki, Finland). During the measurement period of this study, the mean daily rainfall was 1.93 mm, and the mean daily temperature was 15.72 °C.

The BIFoR FACE facility is comprised three experimental treatments across nine experimental arrays, each 30 m in diameter. The treatments are three “elevated CO_2_” arrays (eCO_2_) maintained at 150 ± 38 ppm above ambient CO_2_, three control arrays exposed to ambient CO_2_ (aCO_2_), and three undisturbed woodland areas without fumigation infrastructure. In the present study, only four of the infrastructure arrays were studied due to logistical constraints related to tree canopy access. The fumigation infrastructure arrays have operated from budburst to leaf fall (early April to November) during daylight hours since April 2017, for full details of the experimental design, see Hart et al. (Hart et al. 2020).

### Leaf reflectance sampling

We recorded the reflectance spectra of adaxial leaf surface of *Q. robur* in the sun exposed canopy of two aCO_2_ and two eCO_2_ arrays from July to October 2023. Leaf reflectance measurements were taken on 25th July 2023, 10th August 2023, 24th August 2023, and 6th October 2023. On each measurement day, approximately 30 leaf spectra were taken per tree from two trees per array (60 spectra per array). In total, 932 leaf spectra were recorded, with 469 from aCO_2_ arrays and 463 from eCO_2_ arrays.

Fully expanded leaves were selected that were in the light exposed crown, avoiding leaves with significant herbivory. From the leaves accessible which met these criteria, every third leaf was sampled until 30 spectra had been taken per tree.

Reflectance spectra were recorded using a high-performance single-beam field spectroradiometer over the range 350–2500 nm using a LC-RP Pro leaf clip with active light source (HR-1024i spectrometer, Spectra Vista Corp, USA). The HR-1024i spectrometer combines three dispersion grating spectrometers which overlap in their wavelength ranges to produce continuous spectra from 350 to 2500 nm. The Very Near Infrared (VNIR) spectrometer measures in the range of 350–1000 nm with a 1.5 nm sampling interval. The first SWIR spectrometer measures in the range of 1000–1890 nm with a 3.8 nm sampling interval. The second SWIR spectrometer measures in the range of 1890–2500 nm with a 2.5 nm sampling interval. The spectrometer was calibrated before use by the NERC Field Spectroscopy Facility. The leaf clip attachment is coupled to the spectroradiometer via a 25° armoured fibre optic. The leaf clip forms a seal around the leaf to exclude external light and is illuminated by an internal lamp. The leaf reflectance is integrated within the field of view, approximately 2 cm diameter. An integrating sphere was not used, this allowed time efficient sampling in the canopy (six times faster) but may lead to overestimation of total reflectance compared to measurements taken with single or double integrating spheres (Hovi et al. 2018). An integration time of 2 s was used for all measurements.

During leaf reflectance measurements, the spectrometer was referenced using the leaf clip’s incorporated reflective standard in the leaf clip every 5 min. The HR-1024i applies automatic dark signal baseline correction to every measurement by taking a dark spectrum before each reflectance measurement.

The leaf clip was attached to the adaxial surface in the centre of each leaf, to the left of the midvein, making sure to avoid smaller veins.

### Hyperspectral processing

High-resolution spectroscopy represents a computational and analytical challenge due to large datasets and autocorrelation between wavelength variables; consequently, machine learning techniques are increasingly employed to identify key features in spectral datasets (Meza Ramirez et al. 2021). We used supervised and unsupervised machine learning analyses across the whole spectra to identify principal components of variation and regions of the spectra that change under eCO_2_, alongside conventional vegetation index analysis.

All leaf reflectance spectra were imported and processed using the SpecDAL package (Lee 2017) in Python 3 (Van Rossum and Drake 2009). The overlapping regions of the spectra produced by the three spectrometers comprising the HR-1024i were stitched to give a continuous spectrum from 350 to 2500 nm. The original reflectance spectra were interpolated to obtain data at 1.0 nm wavelength intervals. Absolute reflectance was calculated by multiplying relative reflectance spectra by the laboratory calibrated reflectance of the leaf clip’s reference standard panel (as calibrated by the NERC Field Spectroscopy Facility, panel manufactured by Spectra Vista Corp, USA).

Analysis of the hyperspectral dataset was carried out in R version 4.3.2 (R Core Team 2023) using the following packages: hyperspec (Beleites and Sergo 2017), hyperSpec.utils (Mayer 2024), readr (Wickham et al. 2024a), dplyr (Wickham et al. 2023), tidyr (Wickham et al. 2024b), factoextra (Kassambara and Mundt 2020), caret (Kuhn 2008), FactoMineR (Lê et al. 2008), tibble (Müller and Wickham 2023), scutr (Ganz 2023), kableExtra (Zhu 2024), stringr (Wickham 2023), and effectsize (Ben-Shachar et al. 2020). The following packages were used for data visualisation: ggplot2 (Wickham 2016), viridis (Garnier et al. 2024), and ggpubr (Kassambara 2023).

### Hyperspectral analysis

We calculated the mean total leaf reflectance for eCO_2_ and aCO_2_ exposed leaves by integrating reflectance across the wavelength range and assessed the effect of CO_2_ treatment via linear mixed effect modelling.

We calculated the first ten principal components of the hyperspectral dataset and evaluated the percentage of variance explained by each component. Principle component analysis is an unsupervised method of dimension reduction. We then analysed the difference in values of the first four components between CO_2_ treatments by visualisation, followed by multivariate mixed effect modelling.

To assess the degree of difference between leaf spectra exposed to elevated versus ambient CO_2_, we tested the ability of a machine learning model to predict CO_2_ treatment from leaf reflectance spectra. The spectral data were pre-processed with centring and scaling. Partial least squares discriminant analysis (PLSDA) models were trained on 70% of the leaf reflectance measurements (evenly split between leaves measured in ambient and elevated arrays), using tenfold cross-validation. PLSDA models were calculated with up to 60 components, and the accuracy of models with different numbers of components was evaluated to select an optimum number of components. The PLSDA model with the optimum number of components was then applied to unseen test spectra, and we evaluated the accuracy of the model’s predictions of which CO_2_ treatment leaves had been exposed to. We used the cumulative wavelength importance to understand which wavelength regions were most important for class separation in the PLSDA model.

### Vegetation indices

We calculated a range of vegetation indices from the leaf reflectance spectra and compared the average vegetation index values of leaves exposed to elevated versus ambient CO_2_ using mixed effect modelling. The names, formulas, and original references of the vegetation indices are listed in Table 1.

**Table 1:** Formula of common vegetation indices calculated from leaf reflectance

Vegetation index	Equation	References
Normalised Difference Vegetation Index	${\text{NDVI}} = \frac{\rho 800 - \rho 670}{{\rho 800 + \rho 670}}$	Rouse et al. (1974)
Modified Chlorophyll Absorption Ratio Index	${\text{MCARI}} = \left[ {\left( {\rho 700 - \rho 670} \right) - 0.2*\left( {\rho 700 - \rho 550} \right)} \right]*\left( {\frac{\rho 700}{{\rho 670}}} \right)$	Daughtry et al. (2000)
Photochemical Reflectance Index	${\text{PRI}} = \frac{\rho 570 - \rho 530}{{\rho 570 + \rho 530}}$	Gamon et al. (1997) Peñuelas et al. (1995a)
Plant Senescence Reflectance Index	${\text{PSRI}} = \frac{\rho 680 - \rho 500}{{\rho 750}}$	Merzlyak et al. (1999)
Normalised Difference Nitrogen Index	${\text{NDNI}} = \frac{{\log \left( {\frac{1}{\rho 1510}} \right) - \log \left( {\frac{1}{\rho 1680}} \right)}}{{\log \left( {\frac{1}{\rho 1510}} \right) + \log \left( {\frac{1}{\rho 1680}} \right)}}$	Serrano et al. (2002), Fourty et al. (1996)
Normalised Difference Lignin Index	${\text{NDLI}} = \frac{{\log \left( {\frac{1}{\rho 1754}} \right) - \log \left( {\frac{1}{\rho 1680}} \right)}}{{\log \left( {\frac{1}{\rho 1754}} \right) + \log \left( {\frac{1}{\rho 1680}} \right)}}$	Serrano et al. (2002), Fourty et al. (1996), Melillo et al. (1982)
Normalised Difference Water Index	${\text{NDWI}} = { }\frac{\rho 860 - \rho 1240}{{\rho 860 + \rho 1240}}$	Gao (1996)
Normalised Phaeophytinisation Quotient Index	${\text{NPQI}} = { }\frac{\rho 415 - \rho 435}{{\rho 415 + \rho 435}}$	Peñuelas et al. (1995b), Barnes et al. (1992), Ronen and Galun (1984)

### Statistical analysis: mixed effects modelling

Our dataset contains a hierarchical structure, individual leaves belong to separate trees, arranged in arrays, which then receive different CO_2_ treatments. We also took measurements over time, introducing a time series element to our dataset. To explicitly include these sources of variation in our analysis of the effect of eCO_2_ on different elements of leaf reflectance, we used linear mixed effects modelling. Mixed effects modelling models the effect of fixed effects (such as CO_2_ treatment) on response variables, as well as modelling the variation in response variable explained by random effects (such as hierarchical structure or time-series). Throughout our analysis we used variations of the model:

$${\text{Re}} {\text{sponse}}\,{\text{variable}}\,\sim {\text{CO}}_{{2}} + (1 |{\text{Tree}}) + (1|{\text{Tree}}:{\text{Date}})$$

The tree ID was used as a random effect throughout because it explained significant amounts of variation, whereas the array number did not explain any further variance when included alongside tree ID. Date of measurement for a given tree also explained variance and so was included as a nested random effect. Model estimation was carried out using Restricted Maximum Likelihood (REML), with Satterthwaite’s approximation for degrees of freedom. Multivariate mixed effects modelling was used to model the response of the first four principal components to CO_2_ treatment.

When comparing CO_2_ treatments, density plots are used instead of traditional box plots to display the data distribution.

## Results

### Integrated leaf reflectance

**Fig. 1:**
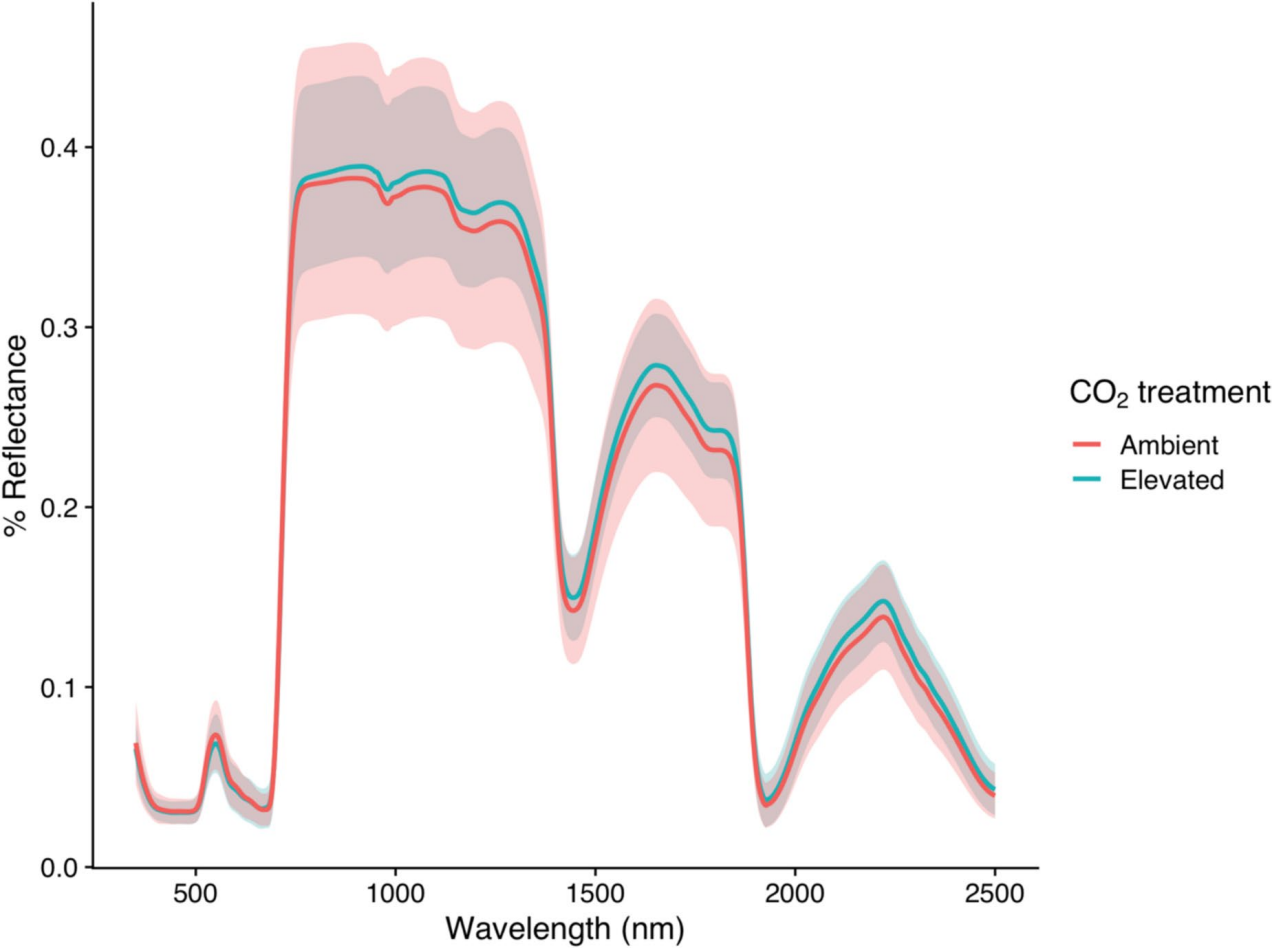
Average percentage leaf reflectance spectra across the range of 350–2500 nm of oak leaves exposed to ambient (red) versus elevated (blue) CO_2_ treatments at the BIFoR FACE experiment. Shaded areas represent ± one standard deviation, with red shading for ambient CO_2_ and blue shading for elevated CO_2_

The average reflectance spectra of leaves exposed to aCO_2_ versus eCO_2_ are visualised in Fig. 1. Visually, the reflectance of leaves exposed to eCO_2_ was higher than those exposed to aCO_2_, except for the green reflectance peak. At the green reflectance peak (540 nm), leaves exposed to eCO_2_ had slightly lower reflectance than leaves exposed to aCO_2_. The position of the Red-Edge does not visually differ between CO_2_ treatments. The infrared reflectance peak between 2200 and 2400 nm was elevated and slightly shifted towards lower wavelengths under eCO_2_.

The mean integrated reflectance for leaves exposed to eCO_2_ was 436.67 (95% CI: 432.40–440.93), whereas under aCO_2_ treatment it was 423.18 (95% CI: 416.34–430.02). Using a linear mixed effects model, the average integrated leaf reflectance under aCO_2_ was an intensity of 423.00 (Standard Error (SE) = 6.93). Under eCO_2_ integrated leaf reflectance was on average 13 units higher or 3.07% (SE = 9.81, Satterthwaite’s degrees of freedom (DF) = 5.97, Satterthwaite’s *P* = 0.23), though this was not statistically significant. Random effects analysis of the date of measurement and tree ID showed that tree-to-tree variability in integrated leaf reflectance accounted for 72.77 variance (Standard Deviation (SD) = 8.53), while date of measurement accounted for 356.67 variance (SD = 18.89). The residual variance in integrated leaf reflectance was 3523.52, indicating large variability which was not explained by CO_2_ treatment, the random effects of tree-to-tree, or date of measurement variability. Inclusion of the array number as a random effect in the model did not explain any further variance.

### Leaf reflectance principal component analysis

**Fig. 2:**
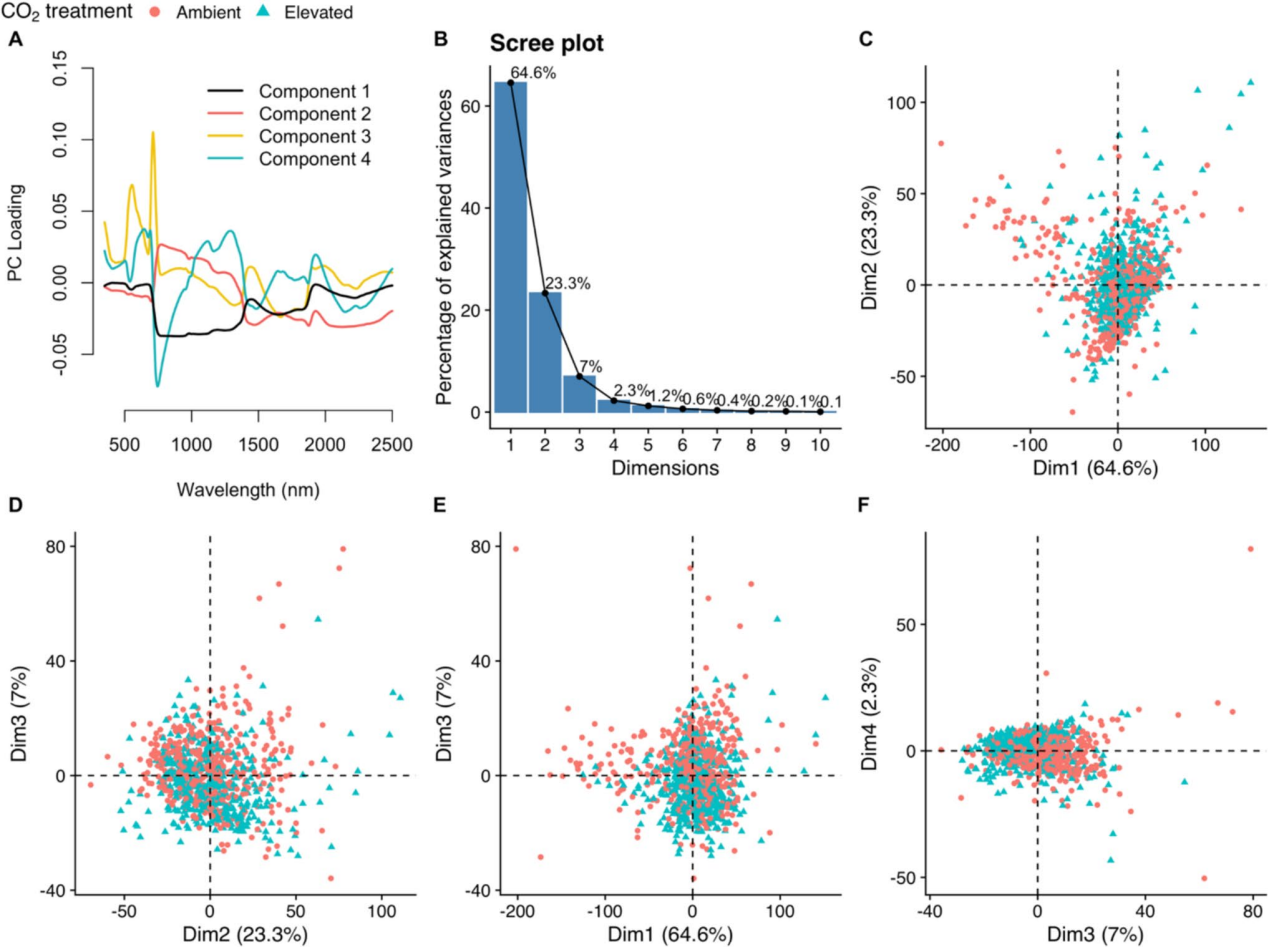
Principal component analysis (PCA) of the leaf reflectance spectra in the range 350–2500 nm of oak trees exposed to ambient (red) versus elevated (blue) CO_2_ treatments at the BIFoR FACE experiment. **a** Wavelength (nm) loading of the first four principal components. **b** Scree plot of variance explained by increasing the number of principal component dimensions. **c**–**f** Leaf reflectance of trees exposed to ambient (red) versus elevated (blue) CO_2_ treatments, plotted against combinations of the first four principal components

**Fig. 3:**
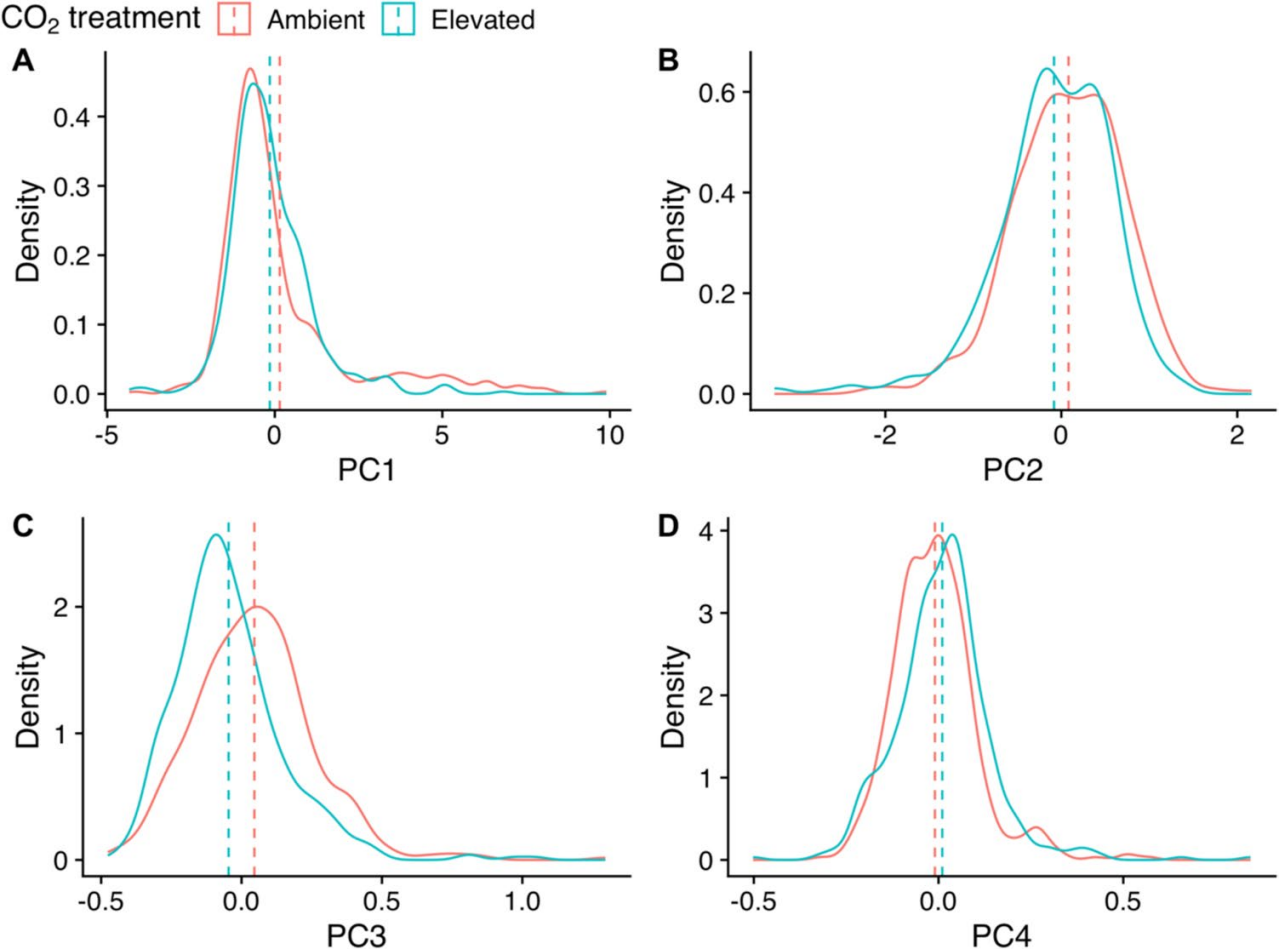
Density plots showing the distribution of values of the first four principal components (PC1-4) in leaf reflectance spectra of oak trees exposed to ambient (red lines) versus elevated (blue lines) CO_2_ treatment. Vertical dashed lines indicate mean principal component value per treatment (ambient in red, elevated in blue). Panel **a** represents principal component 1, panel **b** represents principal component 2, panel **c** represents principal component 3, and panel **d** represents principal component 4. Data taken from mature *Quercus robur* trees at the BIFoR FACE experiment, Staffordshire

The first four principal components (PCs) cumulatively explained 97.2% of the total variance in hyperspectral leaf reflectance (Fig. 2b, PC1: 64.66%, PC2: 23.33%, PC3: 7.04%, PC4: 2.31%). The first component (PC1) is loaded negatively loaded by wavelengths around the red edge (Fig. 2a), while the second component (PC2) is positively loaded by the red edge and wavelengths between 750 and 1500 nm. The third principal component (PC3) is strongly positively loaded by wavelengths characterising the green peak (550 nm) and red edge (680–750 nm), and the fourth component (PC4) is strongly negatively loaded by wavelengths characterising the red edge (680–750 nm) and positively loaded by longer wavelengths. When individual leaf reflectance spectra are plotted against combinations of the first four principal components (Fig. 2c–f), the spectra show limited clustering by CO_2_ dosage. Clustering by CO_2_ dosage is clearer against components two and three (Fig. 2d), and three and four (Fig. 2f). As an unsupervised dimensionality reduction method, principal component analysis reveals that CO_2_ treatment has a limited diverging effect on values of the first four principal components of leaf reflectance. Figure 3 shows the difference in density distribution of principal component scores between CO_2_ treatments, the clearest difference is in component three which has a narrower spread of lower scores under elevated CO_2_ (Fig. 3c).

To assess whether CO_2_ treatment significantly influenced leaf reflectance spectra as summarised by the first four principal components, we fitted a multivariate linear mixed‐effects model with CO_2_ treatment as a fixed effect and random intercepts for tree ID and nested date of measurement.

The model had an intercept of 0.066 (SE = 0.063) for ambient CO_2_ and the fixed effect of eCO_2_ treatment was estimated at − 0.1324 (SE = 0.089). However, this difference was not statistically significant (DF = 5.9, *P* = 0.19). Random effects analyses indicated low between‐group variation. The tree-to-tree variance was 0.01 (SD = 0.10) and among the tree‐by‐date combinations 0.016 (SD = 0.13), while the residual variance (i.e., variation among individual leaves) was substantially larger (0.79; SD = 0.89). Adding the array as an additional level of random effect did not explain any further variation in the principal components of leaf reflectance spectra.

### Leaf reflectance partial least squares discriminant analysis (PLSDA)

**Fig. 4:**
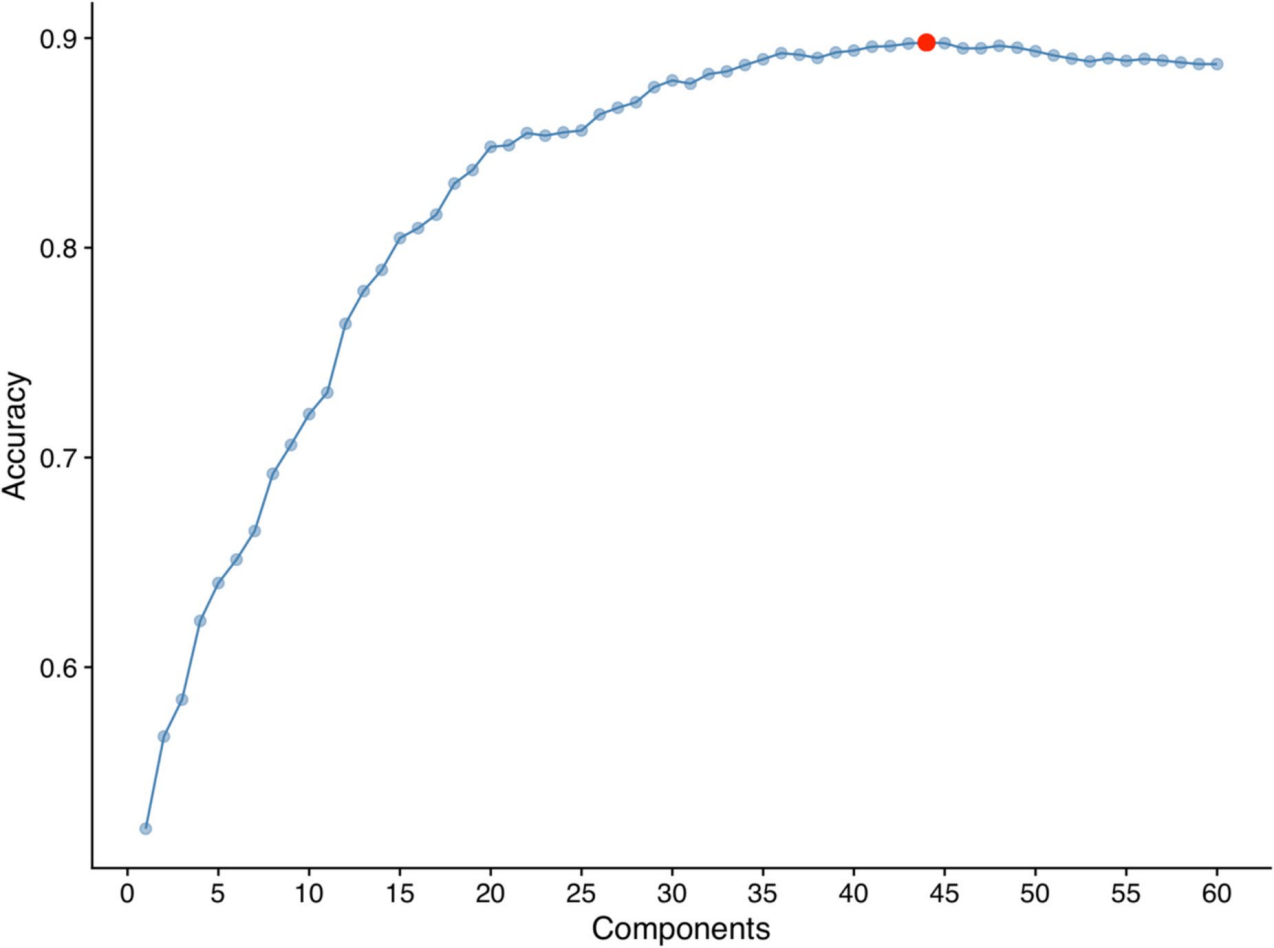
Cross-validation tuning of a partial least squares discriminant analysis (PLSDA) model to separate leaves exposed to elevated versus ambient CO_2_ by their reflectance spectra. Model accuracy is plotted against number of model components, with the optimum number of model components for maximum accuracy highlighted as a red circle (44 components, 0.89 accuracy)

**Fig. 5:**
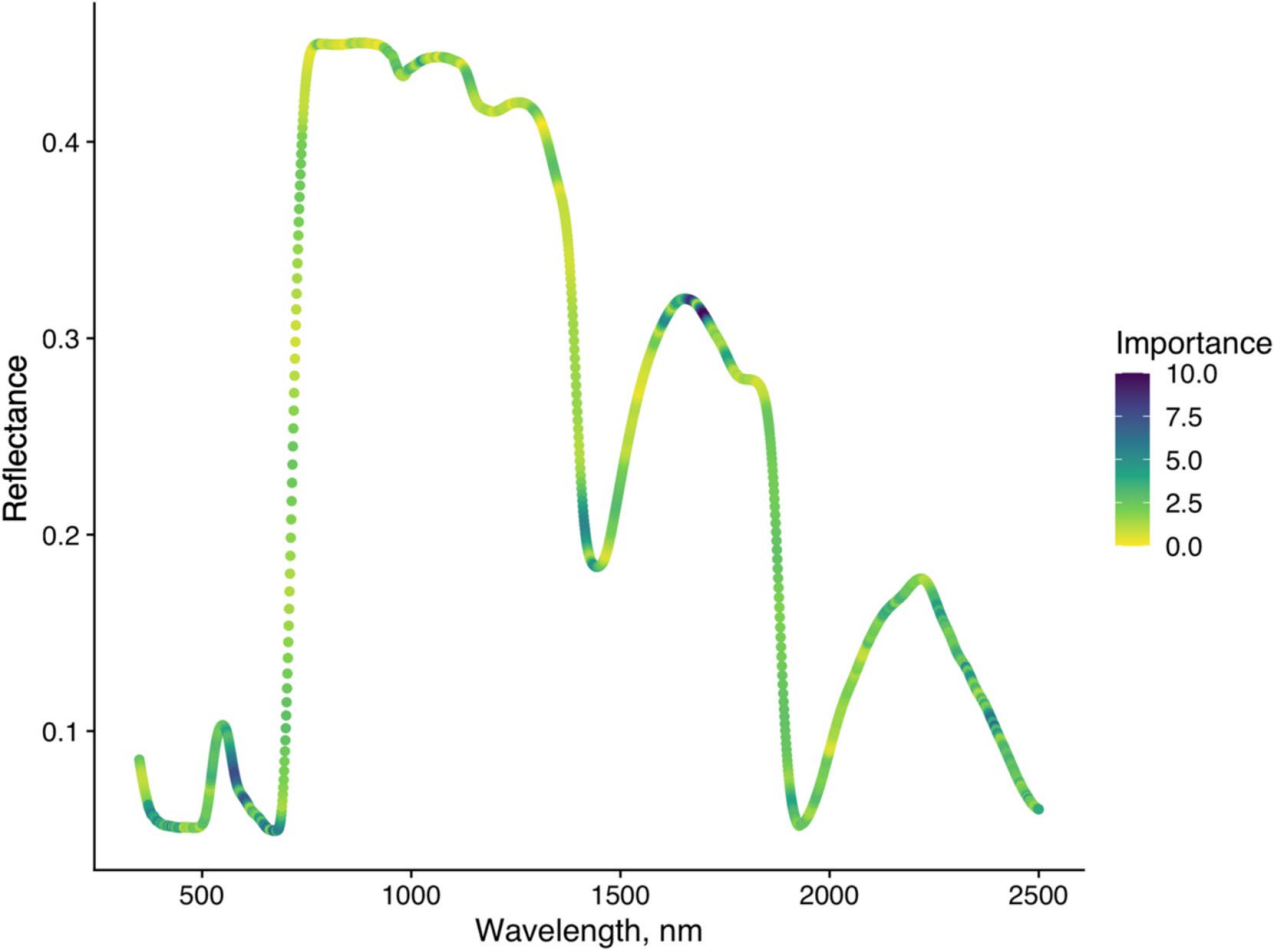
Cumulative wavelength (nm) importance for class separation of CO_2_ treatments (ambient CO_2_ versus elevated CO_2_) by leaf reflectance spectra in partial least squares discriminant analysis (PLSDA) model. Colours indicate the importance of each wavelength to the discrimination model

Tenfold cross-validation of the PLSDA model on the training dataset had the highest accuracy with 44 components (Fig. 4, accuracy = 0.89). The 44-component PLSDA model had an accuracy of 0.88 (95% confidence intervals: 0.84, 0.92) for assigning unknown leaf spectra to the correct CO_2_ treatment, sensitivity was 0.89, and specificity was 0.87. Compared to the ‘no information rate’ (assigning all spectra to aCO_2_ class), the PLSDA model was significantly more accurate (‘no information rate’ accuracy = 0.50, *P* < 2e−16). Between the two classes (aCO_2_ and eCO_2_), error rates were not significantly different (McNemar’s Test, *P* = 0.73). Figure 5 visualises the cumulative importance of different wavelengths in separating the reflectance spectra of aCO_2_ leaves from eCO_2_ leaves. Key regions of importance include the upper edge of the green reflectance peak (565–580 nm), the water absorption region at 1400 nm, and the shortwave infrared peak at 1600–1720 nm. The 44-component PLSDA model was able to accurately separate leaf spectra exposed to aCO_2_ from those exposed to eCO_2_.

### Vegetation indices

**Fig. 6:**
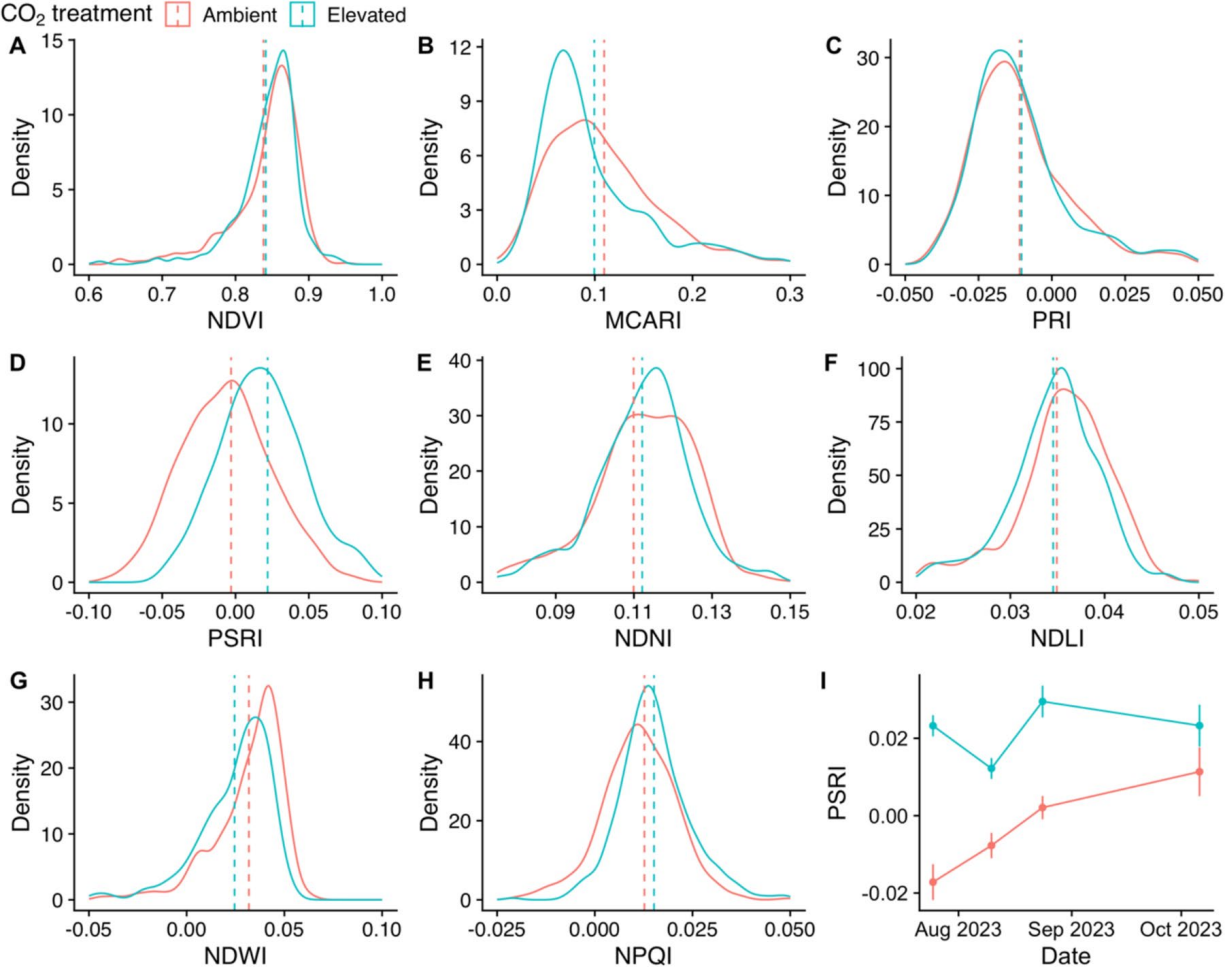
**a**–**h** Density plots showing the distribution of values of range of vegetation indices of oak leaves exposed to ambient (in red) or elevated (in blue) CO_2_ treatment. Dashed lines indicate the mean value of each vegetation index for each CO_2_ treatment. **i** Time series of mean Plant Senescence Reflectance Index (PSRI) for each CO_2_ treatment

Linear mixed‐effects models were fitted to examine the impact of CO_2_ treatment on each vegetation index, with random intercepts for tree and for tree-by-date to account for the hierarchical structure. The results of these models are presented in Table 2. CO_2_ treatment did not have a significant effect on the value of most of the vegetation indices tested except PSRI. Random effect analysis revealed moderate tree-to-tree variation in all vegetation indices, and moderate variation between dates of measurement, although the majority of variation resided at the residual level indicating the predominant source of variation in most vegetation indices in this experiment was at the leaf level and was not affected by CO_2_ treatment. Figure 6 visualises the distributions of vegetation index values of leaves exposed to ambient or elevated CO_2_. Although MCARI did not differ significantly between CO_2_ treatments, the distribution of values under eCO_2_ is much narrower than under aCO_2_ indicating reduced variance in MCARI values under eCO_2_. Similarly, there is a narrower distribution of values around the median NDNI and NPQI values in leaves exposed to eCO_2._

**Table 2:** Summary of linear mixed effect model results for vegetation indices of leaves exposed to ambient (aCO_2_) or elevated CO_2_ (eCO_2_)

Index	aCO_2_		eCO_2_ effect		*T* value	*P* value	Tree variance		Date variance		Residual variance		Figure
	Mean value	SE	Mean effect	SE			Value	SD	Value	SD	Value	SD	
NDVI	8.4 × 10^−1^	7.9 × 10^−3^	+ 4.1 × 10^−3^	1.1 × 10^−2^	3.5 × 10^−1^	7.4 × 10^−1^	1.9 × 10^−4^	1.4 × 10^−2^	1.9 × 10^−4^	1.4 × 10^−2^	2.8 × 10^−3^	5.3 × 10^−2^	6a
MCARI	1.1 × 10^−1^	1.6 × 10^−2^	− 1.0 × 10^−2^	2.2 × 10^−2^	− 4.8 × 10^−1^	6.5 × 10^−1^	6.6 × 10^−4^	2.6 × 10^−2^	1.1 × 10^−3^	3.3 × 10^−2^	2.3 × 10^−3^	4.8 × 10^−2^	6b
PRI	− 1.1 × 10^−2^	4.5 × 10^−3^	+ 5.1 × 10^−4^	6.4 × 10^−3^	8.1 × 10^−2^	9.4 × 10^−1^	6.8 × 10^−5^	8.3 × 10^−3^	4.2 × 10^−5^	6.4 × 10^−3^	2.5 × 10^−4^	1.6 × 10^−2^	6c
PSRI	− 3.1 × 10^−3^	7.3 × 10^−3^	+ 2.6 × 10^−2^	1.0 × 10^−2^	2.5 × 10^−0^	4.9 × 10^−2^ *	1.4 × 10^−4^	1.2 × 10^−2^	2.4 × 10^−4^	1.5 × 10^−2^	1.8 × 10^−3^	4.3 × 10^−2^	6d
NDNI	1.1 × 10^−1^	4.7 × 10^−3^	+ 2.8 × 10^−3^	6.7 × 10^−3^	4.2 × 10^−1^	6.9 × 10^−1^	7.7 × 10^−5^	8.8 × 10^−3^	3.8 × 10^−5^	6.1 × 10^−3^	2.3 × 10^−4^	1.5 × 10^−2^	6e
NDLI	3.5 × 10^−2^	1.5 × 10^−3^	− 3.1 × 10^−4^	2.2 × 10^−3^	− 1.4 × 10^−1^	8.9 × 10^−1^	8.3 × 10^−6^	2.9 × 10^−3^	3.0 × 10^−6^	1.7 × 10^−3^	2.5 × 10^−5^	5.0 × 10^−3^	6f
NDWI	3.1 × 10^−2^	4.7 × 10^−3^	− 7.0 × 10^−3^	6.6 × 10^−3^	− 1.1 × 10^−0^	3.3 × 10^−1^	6.8 × 10^−5^	8.2 × 10^−3^	6.0 × 10^−5^	7.7 × 10^−3^	5.0 × 10^−4^	2.2 × 10^−2^	6g
NPQI	1.3 × 10^−2^	1.7 × 10^−3^	+ 2.0 × 10^−3^	2.4 × 10^−3^	7.6 × 10^−0^	8.3 × 10^−1^	4.1 × 10^−6^	2.0 × 10^−3^	2.5 × 10^−5^	5.0 × 10^−3^	1.3 × 10^−4^	1.2 × 10^−2^	6h

**Note(s):** In all model the degrees of freedom were 5.97, using Satterthwaite’s method. *P* values marked with * indicate a statistically significant effect (*α* = 0.05). All values are given to two significant figures in standard form

PSRI was significantly higher in leaves exposed to eCO_2_ compared to aCO_2_ (see Table 2, + 2.6 × 10^2^ PSRI relative to aCO_2,_ *t* (5.97) = 2.5, *P* = 0.049). Random effects analysis revealed moderate variability among trees (variance = 0.14, SD = 0.012) and dates (variance = 0.24, SD = 1.5 × 10^−4^), although most of the variation resided at the residual level (variance = 1.8 × 10^−3^, SD = 0.043). Leaves exposed to eCO_2_ exhibited higher PSRI values throughout the measurement period, whereas the PSRI of leaves exposed to aCO_2_ increased steadily from July to October. Figure 6i shows how the average PSRI per CO_2_ treatment changed over time.

## Discussion

Our analysis shows that most regions of mature *Q. robur* leaf reflectance spectra and derived vegetation indices were not significantly affected by eCO_2_ treatment after 7 years of eCO_2_ exposure, except in the case of PSRI which was higher in eCO_2_ exposed leaves.

In our study, leaves exposed to eCO_2_ showed a non-significant trend of increased the integrated leaf reflectance, 3.2% higher than leaves exposed to aCO_2_. Leaf reflectance measurements taken with an integrating sphere give a more accurate measurement of total leaf reflectance and any albedo effects by including all possible angles of reflectance, however, when taking many measurements in the forest canopy the speed and practicality of leaf clip reflectance measurements outweighed any benefits an integrating sphere may have offered. Thomas et al. who found a significant leaf reflectance under eCO_2_ in saplings of tropical tree species using an integrated sphere, although the extent of reflectance increase varied from 9% to 23% by species (Thomas 2005). It has been suggested that such changes could affect forest albedo (Thomas 2005). Forest albedo, which refers to the amount of solar radiation reflected back to the atmosphere by forests, plays a critical role in regulating the Earth’s climate and is a source of uncertainty in the radiation budget of climate models (Alibakhshi et al. 2020). Forest albedo is influenced not only by forest structure but also by leaf-level optical properties (Hollinger et al. 2010; Meunier et al. 2022; Henniger et al. 2023). For example, in China, forest greening between 2002 and 2019 was accompanied by an increase in shortwave albedo (Yan et al. 2021). Luyssaert et al. highlighted the lack of consideration of changes to forest albedo in the management of forests aimed at offsetting CO_2_ emissions (Luyssaert et al. 2018). Despite this, the effect of eCO_2_ on forest albedo remains understudied. Our results suggest that in the long-term eCO_2_ may not have a significant effect on the total leaf reflectance of mature *Q. robur*, but more detailed analysis of total leaf reflectance and transmission using an integrating sphere would be necessary to predict the affect of eCO_2_ on oak forest albedo. Further analysis is needed to understand how the total reflectance of different mature tree species will respond to future CO_2_ concentrations, as well as the effect on temperate forest albedo.

As hypothesised, the spectral reflectance profile of leaves exposed to eCO_2_ was similar to that of leaves exposed to aCO_2_. The spectral signature as summarised by the first four principal components was not significantly affected by CO_2_ treatment after accounting for hierarchical structure. The large residual in the mixed effects model of principal components suggests that most of the variation in leaf reflectance spectra arose at the leaf level rather than at the tree or temporal level. Most of the vegetation indices examined also did not respond to CO_2_ treatment, although the reduced spread of MCARI, NDNI, and NPQI values under eCO_2_ could indicate some form of directional response. The majority of previous studies which have examined the effect of eCO_2_ on the leaf reflectance of trees have also not found a significant eCO_2_ effect in mature *Quercus pubescens* (Stylinski et al. 2000), in young *Acer saccharum* (Carter et al. 2000), in mature *Liquidambar styraciflua* (Wicklein et al. 2012), nor in mature *Eucalyptus tereticornis* (Wujeska-Klause et al. 2019). Significantly increased reflectance in the green peak and red edge was reported in *Pinus strobus L* exposed to eCO_2_ (Carter et al. 1995). eCO_2_-induced spectral changes have also been reported in crops (Gray et al. 2010; Tormena et al. 2019). On balance, the lack of eCO_2_ response in the spectral profile of *Q. robur* in this study is in keeping with previous research in deciduous tree species. This suggests the spectral profile of future deciduous forests will not be broadly altered by future CO_2_ concentrations.

Our PLSDA model is useful in highlighting the regions of the spectra which differed most between CO_2_ treatments and so answering the question “how does eCO_2_ affect leaf reflectance?” in a nuanced way. The key regions of differentiation were the upper edge of the green reflectance peak, the water absorption region at 1400 nm, and the shortwave infrared peak around 1650 nm. First, the green reflectance peak is affected by the overlapping reflectance peaks of different chlorophyll, carotene, and anthocyanin pigments (Sims and Gamon 2002), so changes in the upper edge green peak reflectance could reflect a change in one or multiple pigment concentrations.

The 1400 nm water absorption peak was also important in differentiating between leaves exposed to different CO_2_ treatments. In a different species of oak (*Quercus agrifolia*), the 1650 nm peak has also been shown to correspond to leaf water status (Pu et al. 2003), but it has not been explicitly characterised in *Q. robur*. In a global meta-analysis, ECO_2_ has been shown to increase leaf water content under drought conditions (Wang et al. 2022). Generally, eCO_2_ has been found to increase vegetation’s water use efficiency (WUE) as a result of stomatal closure and/or increased photosynthetic activity (Gilbert et al. 2011; Gardner et al. 2023). Additionally, in the BIFoR FACE experiment, there were no significant changes to water use under eCO_2._

Wavelengths in the 1650 nm reflectance peak were the most important in the PLSDA model’s differentiation between CO_2_ treatments. Tormena et al. (2019) also identified a response to eCO_2_ in coffee leaf reflectance bands around 1657 nm and 1698 nm via PLSDA (Tormena et al. 2019). The 1650 nm peak is an indirect reflectance band, due to chemicals such as cellulose (Curran 1989) and affected by leaf water content (DANSON et al. 1992; Tian et al. 2001). The cell wall hemicellulose content increased in *Betula pendula* leaves exposed to eCO_2_ (Oksanen et al. 2005), but the effect of eCO_2_ on leaf cellulose content in oak species has not been well examined. More broadly, eCO_2_ has been shown to increase leaf carbohydrate content (Kinney et al. 1997; Agrell et al. 2000; Coley et al. 2002), and at the BIFoR FACE experiment foliar carbon content in fresh leaves was significantly higher under eCO_2_ (Roberts et al. 2022), which could include changes to cellulose content. While firm conclusions cannot be drawn from the PLSDA analysis, the importance of wavelengths in the upper green reflectance, water absorption band, and 1650 nm peak are useful to highlight as directions for future investigation of the foliar effects of eCO_2_ in mature deciduous trees.

The one vegetation index which CO_2_ treatment had a significant effect on was PSRI. PSRI is sensitive to the ratio of carotene to chlorophyll pigments in the leaf and is used as a quantitative measure of senescence-induced degradation of chlorophyll (Merzlyak et al. 1999). PSRI was significantly higher under eCO_2_ and was elevated early in the growing season compared to trees exposed to aCO_2_. This indicates changes in the relative content of carotenes and chlorophyll under eCO_2_. Since no changes in chlorophyll content have been found with eCO_2_ treatment at BIFoR FACE (Gardner et al. 2022b), the increase in PSRI is more likely to be due to increased carotene content than decreased chlorophyll. Reductions in carotene content with eCO2 treatment have been found previously (Loladze et al. 2019). In the only study on *Q. robur*, the decrease in carotenoid content under eCO_2_ was not significant (Schwanz and Polle 2001), but this study was on seedlings in pots so may not be transferrable to adult trees in a mature forest. Norby et al. (2024) reported leaf loss started earlier in some years under eCO_2_ at the BIFoR FACE experiment, which could also lead to earlier chlorophyll breakdown and elevated PSRI (Norby et al. 2024). Other FACE experiments have also reported extended senescence (Sigurdsson 2001), although in poplar senescence was delayed by eCO_2_ (Cotrufo et al. 2005; Taylor et al. 2008). In tree seedling experiments white birch and basswood, autumn senescence accelerated under eCO_2_ (Li et al. 2019; Tedla et al. 2020). Ontogeny is easier to study in herbaceous plants, but there are also mixed effects of eCO_2_ on herbaceous plant senescence (Curtis et al. 1989; de la Mata et al. 2012). We would encourage future work to examine the pigment concentrations of leaves from the BIFoR FACE experiment via HPLC, to quantify the changes in carotenes indicated by our spectral data and how pigments change seasonally in relation to senescence.

In large scale ecosystem manipulation experiments, like the BIFoR FACE experiment, engineering and financial constraints limit the number of replicate arrays and thus limit the “n” of the study. With small numbers of replicate arrays, very large changes are needed between treatments to produce statistically significant results using traditional statistical methods (Norby et al. 2024). Mixed effect modelling goes someway to address this issue. However, the value of observations from large ecosystem manipulation experiments should not be dismissed on account of the difficulty in producing statistically significant results while avoiding pseudo-replication (Davies and Gray 2015).

## Conclusion

Long-term exposure to eCO_2_ did not significantly change the intensity of leaf reflectance or the overall spectra of mature *Q. robur* trees. However, the Plant Senescence Reflectance Index (PSRI) increased under eCO_2_ conditions, indicating a change in the ratio of chlorophyll to carotene pigments. Our analysis also highlighted reflectance changes in the infrared spectrum, which may correspond to alterations in leaf water content and carbohydrate content. Hyperspectral leaf reflectance is a useful tool for understanding the consequences of future atmospheric CO₂ concentrations on foliar features such as pigments.

## Data Availability

The spectral dataset and analysis code used in this research will be freely available to download at https://doi.org/10.5281/zenodo.15323740 upon publication.
